# Morphological characterization, genetic diversity and population structure of the rice blast pathogen *Magnaporthe oryzae* in Northeast India

**DOI:** 10.1038/s41598-026-51508-9

**Published:** 2026-06-30

**Authors:** Mathew Seikholen Baite, Letngam Touthang, Benjamin Chungjapao Haokip, Tasvina R. Borah

**Affiliations:** 1 Division of Crop Science, ICAR Research Complex for NEH Region, Umiam, Meghalaya, 793103 India; 2 Department of Molecular Biology and Biotechnology, Rani Lakshmibai Central Agricultural University, Jhansi, Uttar Pradesh India

**Keywords:** Genetic diversity, *Magnaporthe oryzae*, Morphological Characterization, Rice Blast, SSR markers, Genetics, Microbiology, Molecular biology, Plant sciences

## Abstract

The blast pathogen, *Magnaporthe oryzae*, is one of the most destructive fungal pathogens of rice worldwide, yet its morphological features, genetic diversity and population structure in Northeast India remain poorly understood. In this study, twenty‒two *M. oryzae* isolates collected from eight states of Northeast India were characterized using morphological, molecular, and population genetic analyses. Morphological characterization revealed whitish to greyish‒white mycelia with sparse sporulation and colony diameters ranged from 36 to 90 mm, classifying the isolates into 14 fast and 8 slow‒growing groups. Whole genome sequencing was performed to enable both ITS‒based identification and SSR locus mining from the assembled genomes. Molecular identification using ITS rDNA sequences confirmed all isolates as *M. oryzae*, with 95.5–100% similarity. Phylogenetic analysis grouped the isolates into two major clades and identified seven ITS sequence types (GenBank Accessions: PX273287–PX273293). Genetic diversity assessed using 30 SSR markers revealed substantial polymorphism, with 1–7 alleles per locus and polymorphism information content (PIC) values ranging from 0.00 to 0.81. Heatmap clustering, dendrogram analysis, and distance metrics consistently identified two major genetic groups, with some isolates forming nearly identical clusters and others showing moderate divergence. Principal Component Analysis (PCA) and Principal Coordinates Analysis (PCoA) accounted for 87.8% of the total variance (PC1 and PC2 accounted for 54.4% and 33.4% respectively of the total variance) and revealed distinct outliers. Analysis of Molecular Variance (AMOVA) attributed 80% of the total genetic variation to differences among populations while only 20% was attributed to within population differences highlighting significant inter‒population divergence and clonal population structure. The study revealed substantial morphological and genetic diversity among *M. oryzae* populations in Northeast India, underscoring the need for region‒specific disease management strategies.

**Supplementary Information:**

The online version contains supplementary material available at https://doi.org/10.1038/s41598-026-51508-9.

## Introduction

Rice blast caused by *Magnaporthe oryzae* is one of the most damaging rice diseases worldwide, infecting more than 50 grass species, including wheat, barley, oats, and millet, and causing yield losses of up to 30%^1^. In severe cases, upto 100% yield loss is also reported due to Rice Blast. The preferred name for the causal organism of rice blast has evolved over time due to advancements in taxonomic classification and nomenclature standards. Earlier, the pathogen of rice blast was called *Pyricularia oryzae* as the asexual or anamorph and *M. oryzae* as the sexual or teleomorph stage. However, under the One Fungus One Name policy that came into effect in January 2013, the pathogen of rice blast was named *Magnaporthe oryzae*, regardless of whether it is identified by its sexual or asexual form.

The leaves, stem, neck, node and panicle of rice are the main areas infected by the pathogen. The symptoms of rice blast are diamond or spindle shaped lesions with a grey or whitish centre and brown to reddish‒brown margins on leaves. The lesions may enlarge and join together, producing drying and burning of leaves. Neck blast is the most destructive form. The infected neck (panicle base) portion is brown or black, broken and hanging, often leading to poor grain filling. The global importance of the pathogen, *M. oryzae* is evident from its position as the top‒ranked pathogen in the *top 10 fungal pathogens in molecular Plant Pathology* list published in Molecular Plant Pathology^2^.

In India, the rice blast pathogen, *M. oryzae*, is well studied in several aspects like morphological description, genetic diversity, population structure, genomic, transcriptomics and proteomics etc. However, limited information is available on the morphological characterization, genetic diversity, and population structure of *M. oryzae* in Northeast India. Understanding the genetic diversity and population structure of the pathogen is key to developing effective strategies for its control^3^. Therefore, the present study aimed to collect, isolate, identify and characterize the morphological structure of *M. oryzae* isolates from Northeast India, confirmed their identification by ITS sequencing and phylogenetic analysis and investigate their genetic diversity using SSR molecular markers. The study will provide crucial insights into the morphological, genetic diversity and population structure of *M. oryzae* isolates, facilitating the development of targeted resistance breeding programs in Northeast India.

## Materials and methods

### Sample collection and isolation

**Fig. 1:**
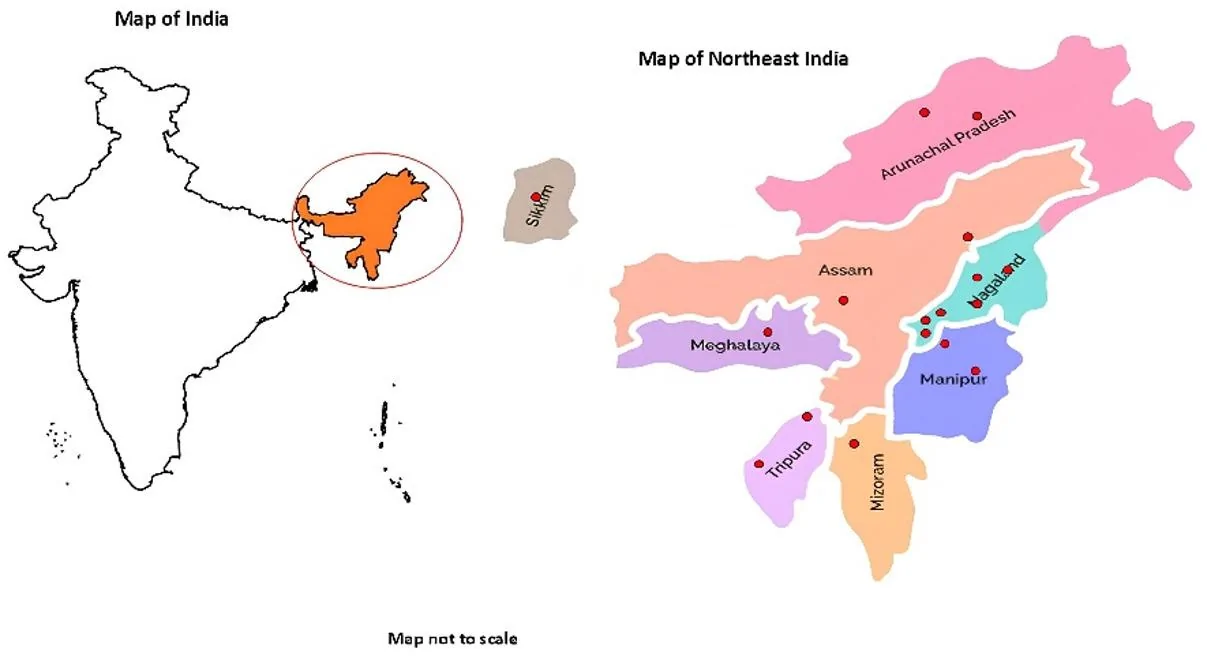
Map showing the locations in Northeast India where rice blast samples were collected.

The rice blast samples were collected from different locations in Northeast India during the wet season (monsoon) of 2023 (Fig. 1).

The pathogen, *M. oryzae* was isolated from the disease samples using artificial media like potato dextrose agar (PDA). The infected rice tissue was washed with 1% sodium hypochlorite for 30 s, rinsed with sterile water and dried using blotting paper. The tissues were cut into small pieces about 1–3 mm in size and using a needle placed into 3 places on a Petri plate forming a triangular arrangement, equidistant from one another. The plates were sealed with parafilm and labelled with name, date of the isolation and incubated at the appropriate temperature (26–28 °C). After 3‒5 days, the germinated mycelium was transferred to new media to obtain pure culture for further study.

### Identification and morphological characterization

The morphological characterization of 22 *M. oryzae* isolates was performed for colony growth diameter and mycelial colour at 6 days after inoculation. For microscopic observation, examination was done under a compound microscope to observe the conidia and hyphae. Each of the *M. oryzae* isolates were grown on potato dextrose broth media using a 500 ml conical flask for over a week to get sufficient quantity of mycelium for DNA Extraction. The mycelial mass was carefully separated from the broth medium, washed with sterile water, and dried using blotting paper for packing under aluminum foil and stored at -80 °C.

### Molecular identification and phylogenetic analysis

Low-pass whole genome sequencing (LP-WGS) using a reference-based sequencing was performed to enable both ITS‒based identification and SSR locus mining from the assembled genomes.

### Genomic DNA extraction and quality assessment

Genomic DNA of *M. oryzae* isolates was extracted manually using the AllPrep Fungal DNA/RNA/Protein Kit (QIAGEN, Cat. No. 47054) following the manufacturer’s protocol, with 50 mg of fungal sample as input and a final elution volume of 100 µL. The concentration and purity of extracted DNA were assessed using a NanoDrop 1000 spectrophotometer (Thermo Fisher Scientific). DNA integrity was verified by electrophoresis on a 2% agarose gel stained with ethidium bromide. Approximately 2 µL of each DNA sample was mixed with 1 µL of 2× loading dye (Invitrogen) and electrophoresed at 80 V for 60 min. A TekBio DNA Ladder (Cat. No. TL20240701) was used as a molecular size marker.

### Library preparation, sequencing and assembly

Library preparation was conducted using the KAPA HyperPlus Kit (Roche, Cat. No. 07962428001) according to the manufacturer’s protocol. Final libraries were quantified with a Qubit 4.0 fluorometer (Thermo Fisher, Cat. No. Q33238) using the Qubit dsDNA HS Assay Kit (Thermo Fisher, Cat. No. Q32851). Insert size distribution was determined using an Agilent 4150 TapeStation with High Sensitivity D1000 ScreenTapes (Agilent, Cat. No. 5067‒5582). The pooled libraries were sequenced on the Illumina NovaSeq 6000 System using an S4 flow cell (Rapid Run Mode, 2 × 150 bp) at Centyle Biotech Pvt. Ltd., New Delhi. Demultiplexing of pooled libraries was performed using BaseSpace™ Sequence Hub (Illumina). Initial quality assessment of raw reads was performed using FastQC. Subsequent quality control and filtering were conducted with fastp to: (a) remove adapter sequences; (b) discard reads with a Phred quality score < 30 in more than 30% of bases; and (c) remove reads shorter than 70 bp. Post‒filtering, the average read length was 159 bp. High‒quality reads were assembled de novo using MEGAHIT.

### Identification and phylogenetic analysis

Internal Transcribed Spacer (ITS) sequences were identified from the assembled genomes via BLASTn search using a reference *M. oryzae* ITS sequence obtained from NCBI GenBank (Accession No. MK595550.1). All detected ITS regions were validated by reconfirmation through NCBI BLASTn. The consensus ITS sequences were generated from forward and reverse reads using Aligner software, and a representative sequence was used for comparative analysis. The confirmed ITS sequence was subjected to a BLASTn search against the NCBI GenBank database to identify closely related isolates. The ten sequences showing the highest identity scores were retrieved for phylogenetic comparison. Multiple sequence alignment was performed using ClustalW, and a distance matrix was generated. The ITS region was sequenced from each isolate and all sequences were aligned to identify nucleotide polymorphisms. Sequences sharing identical nucleotide patterns across the ITS alignment were grouped together and defined as a single haplotype, while sequences differing at one or more positions were considered different sequence types. Based on these sequence differences, seven distinct ITS sequence types were identified. Phylogenetic trees were constructed using the Maximum Likelihood method implemented in MEGA X. Taxonomic annotation was further validated by comparison with the NCBI non-‒redundant nucleotide (nt) database.

### Genetic diversity analysis

SSR loci were identified in silico from assembled genomes based on primer binding and predicted amplicon sizes, and alleles were scored computationally.

### Genome assembly and SSR marker identification

High‒quality filtered reads were assembled into contigs using the MEGAHIT genome assembler, which employs a de Bruijn graph algorithm to reconstruct genome sequences from short‒read data based on sequence overlaps. Genome assembly was performed independently for each of the 22 *M. oryzae* isolates. The resulting assemblies were screened for 30 Simple Sequence Repeats (SSRs) (Supplementary Table 1) using the PrimerSearch tool from the EMBOSS suite.

Forward and reverse primer sequences were queried against each assembled genome to identify SSR loci. The search parameters were set as follows: (a) a maximum of three mismatches allowed between primer and target sequences; and (b) an expected PCR product length ranging from 100 to 600 base pairs. The SSR loci identified from the assemblies were subsequently used for downstream genetic diversity and comparative analyses.

### Polymorphic information content (PIC)

To measure the informativeness of the markers, the polymorphism information content (PIC) was calculated using the formula suggested by Weir 1996^4^ as below:

  

$$PIC=1 - \sum\limits_{{i=1}}^{n} {P_{i}^{2}}$$

where “i” is the total number of alleles detected for the SSR marker and “Pi” is the frequency of the ith plus allele.

### Population structure analysis

The genetic relationships among 22 *M. oryzae* isolates (MO1–MO22) were examined using 30 polymorphic Simple Sequence Repeat (SSR) markers. Pairwise genetic distances were computed to construct a genetic distance matrix and visualized as a heatmap to assess inter‒isolate similarity. The Unweighted Pair Group Method with Arithmetic Mean (UPGMA) hierarchical clustering based on a dissimilarity matrix was computed in the Pheatmap package in R‒studio version 4.2.2^5^. The overall genetic structure was further assessed using Principal Component Analysis (PCA) and Principal Coordinates Analysis (PCoA) to detect patterns of clustering and genetic divergence. Population differentiation was quantified through Analysis of Molecular Variance (AMOVA), which partitioned total genetic variation into components within and among populations. The AMOVA and principal coordinate analysis (PCoA) were computed in the GenAlEx 6.51b2 version^6^. Descriptive statistics, including mean pairwise distance (MPD) and mean nearest taxon distance (MNTD), were calculated to estimate the degree of relatedness among isolates. Boxplots and histograms of genetic distance distributions were generated to assess the overall genetic diversity and frequency distribution patterns within the *M. oryzae* population.

## Results

### Identification and morphological characterization

**Fig. 2:**
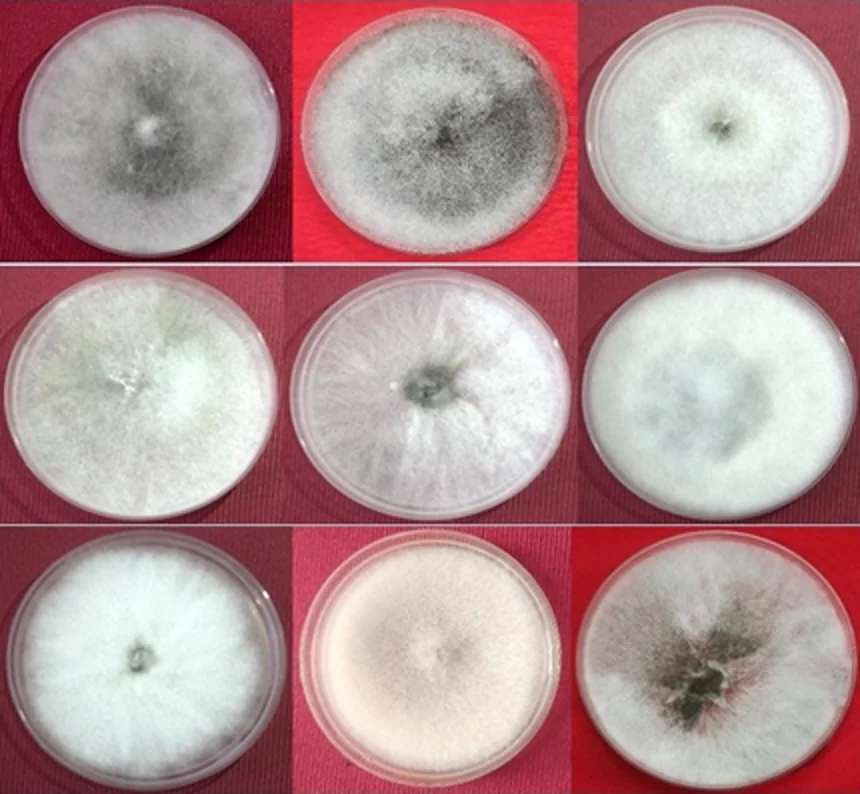
Colony morphology of *Magnaporthe oryzae* isolates grown on PDA, illustrating natural phenotypic variation.

The pathogen, *M. oryzae* produced whitish to greyish‒white mycelial masses when cultured on Petri dishes, which are characteristic of the species (Fig. 2).

Colony diameters among the 22 isolates ranged from 36 mm to 90 mm, with a mean of 75.27 mm. Based on colony growth diameter, the isolates were grouped into two categories: Fast Growth (≥ 75.27 mm) comprising 14 isolates (MO1, MO2, MO3, MO4, MO5, MO7, MO11, MO12, MO13, MO14, MO15, MO16, MO20, and MO22), and Slow Growth (< 75.27 mm) consisting of 8 isolates (MO6, MO8, MO9, MO10, MO17, MO18, MO19, and MO21). Sporulation was generally sparse, with only a few isolates producing conidia, while most failed to sporulate. The conidium was three‒celled and pyriform‒shaped. The isolates exhibited three main mycelial colour variations‒greyish white, greyish, and whitish. Accordingly, they were grouped into two colour‒based categories: Group 1 (Greyish‒type mycelium) comprising 18 isolates (MO1, MO2, MO3, MO4, MO5, MO6, MO8, MO9, MO11, MO12, MO13, MO14, MO15, MO16, MO17, MO19, MO21, and MO22), and Group 2 (Whitish mycelium) consisting of 4 isolates (MO7, MO10, MO18, and MO20) (Table 1).

**Table 1:** Source of *Magnaporthe oryzae* isolates and their morphological characterization.

Isolate	Place of collection	State	Colony growth diameter (mm)	Group	Mycelial colour
MO1	Bade	Nagaland	80.00	Fast Growth	Greyish white
MO2	Umiam‒1	Meghalaya	80.00	Fast Growth	Greyish white
MO3	Jharnapani	Nagaland	90.00	Fast Growth	Greyish white
MO4	Dharmanagar	Tripura	90.00	Fast Growth	Greyish white
MO5	Jalukie	Nagaland	90.00	Fast Growth	Greyish white
MO6	Peren	Nagaland	50.00	Slow Growth	Greyish white
MO7	Maova	Nagaland	80.00	Fast Growth	Whitish
MO8	Umiam‒2	Meghalaya	70.00	Slow Growth	Greyish
MO9	Ranipool	Sikkim	50.00	Slow Growth	Greyish white
MO10	Kathiatoli	Assam	65.00	Slow Growth	Whitish
MO11	Manza	Assam	90.00	Fast Growth	Greyish white
MO12	Lembucherra‒1	Tripura	90.00	Fast Growth	Greyish
MO13	Lembucherra‒2	Tripura	90.00	Fast Growth	Greyish white
MO14	Jorhat	Assam	90.00	Fast Growth	Greyish white
MO15	Zunheboto	Nagaland	90.00	Fast Growth	Greyish white
MO16	Palin	Arunachal Pradesh	80.00	Fast Growth	Greyish white
MO17	Tujang Waichong	Manipur	65.00	Slow Growth	Greyish white
MO18	Kohima	Nagaland	36.00	Slow Growth	Whitish
MO19	Baghty	Nagaland	50.00	Slow Growth	Greyish white
MO20	Kolasib	Mizoram	80.00	Fast Growth	Whitish
MO21	Kshetrigao	Manipur	70.00	Slow Growth	Greyish white
MO22	Basar	Arunachal Pradesh	80.00	Fast Growth	Greyish white
		Min	36		
		Max	90		
		Mean	75.27		
		Std. error	3.47		
		Stand. dev	16.32		
		Coeff. var	21.68		

Two representative pure cultures of *M. oryzae* were deposited to The Indian Type Culture Collection (ITCC), New Delhi having the following accession numbers: ITCC 9553 and ITCC 9554.

### Molecular identification and phylogenetic analysis

The ITS sequencing and BLAST findings of all 22 samples verified precise species identification with the top hits corresponding to *M. oryzae*. With E‒values of 0 and high query coverage (99‒100%), each isolate showed accurate and dependable matching (Table 2).

**Table 2:** ITS of 22 *Magnaporthe oryzae* isolates producing significant alignments.

Sample	Scientific Name	Query cover (%)	E value	Percentage identity
MO1	*Magnaporthe oryzae*	100	0	100
MO2	*Magnaporthe oryzae*	100	0	100
MO3	*Magnaporthe oryzae*	100	0	100
MO4	*Magnaporthe oryzae*	100	0	100
MO5	*Magnaporthe oryzae*	100	0	97.84
MO6	*Magnaporthe oryzae*	100	0	100
MO7	*Magnaporthe oryzae*	100	0	100
MO8	*Magnaporthe oryzae*	100	0	99.8
MO9	*Magnaporthe oryzae*	100	0	100
MO10	*Magnaporthe oryzae*	99	0	99.61
MO11	*Magnaporthe oryzae*	100	0	95.53
MO12	*Magnaporthe oryzae*	100	0	100
MO13	*Magnaporthe oryzae*	100	0	100
MO14	*Magnaporthe oryzae*	100	0	100
MO15	*Magnaporthe oryzae*	100	0	97.84
MO16	*Magnaporthe oryzae*	100	0	97.84
MO17	*Magnaporthe oryzae*	100	0	97.84
MO18	*Magnaporthe oryzae*	100	0	100
MO19	*Magnaporthe oryzae*	100	0	97.84
MO20	*Magnaporthe oryzae*	100	0	100
MO21	*Magnaporthe oryzae*	100	0	97.84
MO22	*Magnaporthe oryzae*	100	0	99.8

Strong species‒level homogeneity was confirmed by the majority of isolates showing 100% similarity to reference sequences. Minor intraspecific variation within the ITS region was suggested by a few isolates with somewhat lower identities (ranging from 95.5% to 99.8%), including MO5, MO10, MO11, MO15, MO16, MO17, MO19, MO21, and MO22. Although values like ~ 95.53% and 97.84% for the ITS region may seem low compared with some barcoding thresholds, fungal ITS sequences commonly show intraspecific variation, and within‒species ITS similarity can range substantially in fungi, meaning matches below strict thresholds can still be consistent with species‒level identification in taxa like *M. oryzae*.

**Fig. 3:**
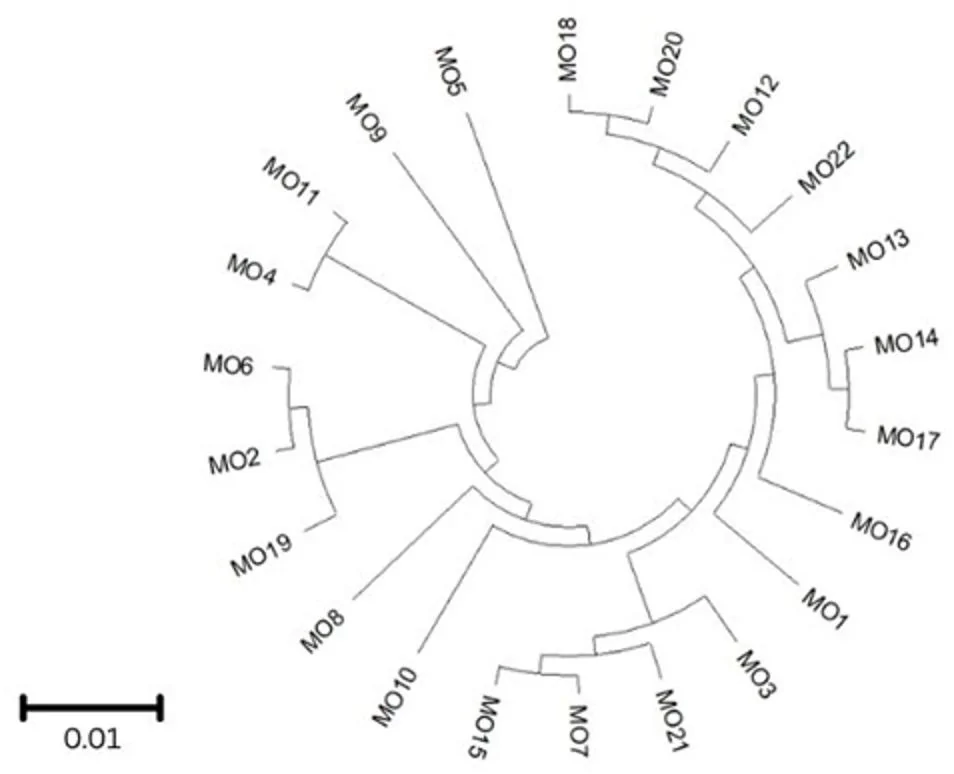
Circular phylogenetic tree showing the genetic relationships among 22 *Magnaporthe oryzae* isolates. The branch lengths specify genetic distance, with shorter branches indicating higher similarity among the fungal isolates.

In the phylogenetic analysis, all the ITS sequences predicted from 22 samples were present in the same clade as that of *M. oryzae* ITS sequences, confirming the identity of 22 samples as *M. oryzae*. The circular phylogenetic tree of the 22 isolates (MO1–MO22) showed two major clusters that separate the isolates into distinct genetic groupings (Fig. 3).

The first major cluster included MO1, MO3, MO16, MO17, MO13, MO14, MO22, MO12, MO20, and MO18, which formed a relatively compact set of branches with short genetic distances, indicating close relatedness. The second major cluster contained MO2, MO6, MO4, MO11, MO9, MO5, MO19, MO8, MO10, MO15, MO7, and MO21, which displayed slightly deeper branching, reflecting greater genetic diversity within the cluster. Notably, MO4, MO6, and MO2 formed a well‒defined sub‒group in this cluster. Overall, the short branch lengths across the tree indicated that all isolates were genetically similar, consistent with variation expected within a single species. The scale bar (0.01) denotes the genetic distance, so branch length correlates with sequence divergence. Based on ITS sequences, seven ITS sequence types of *M. oryzae* were identified which were deposited in GenBank under accession numbers PX273287 – PX273293.

### Genetic diversity analysis

**Fig. 4:**
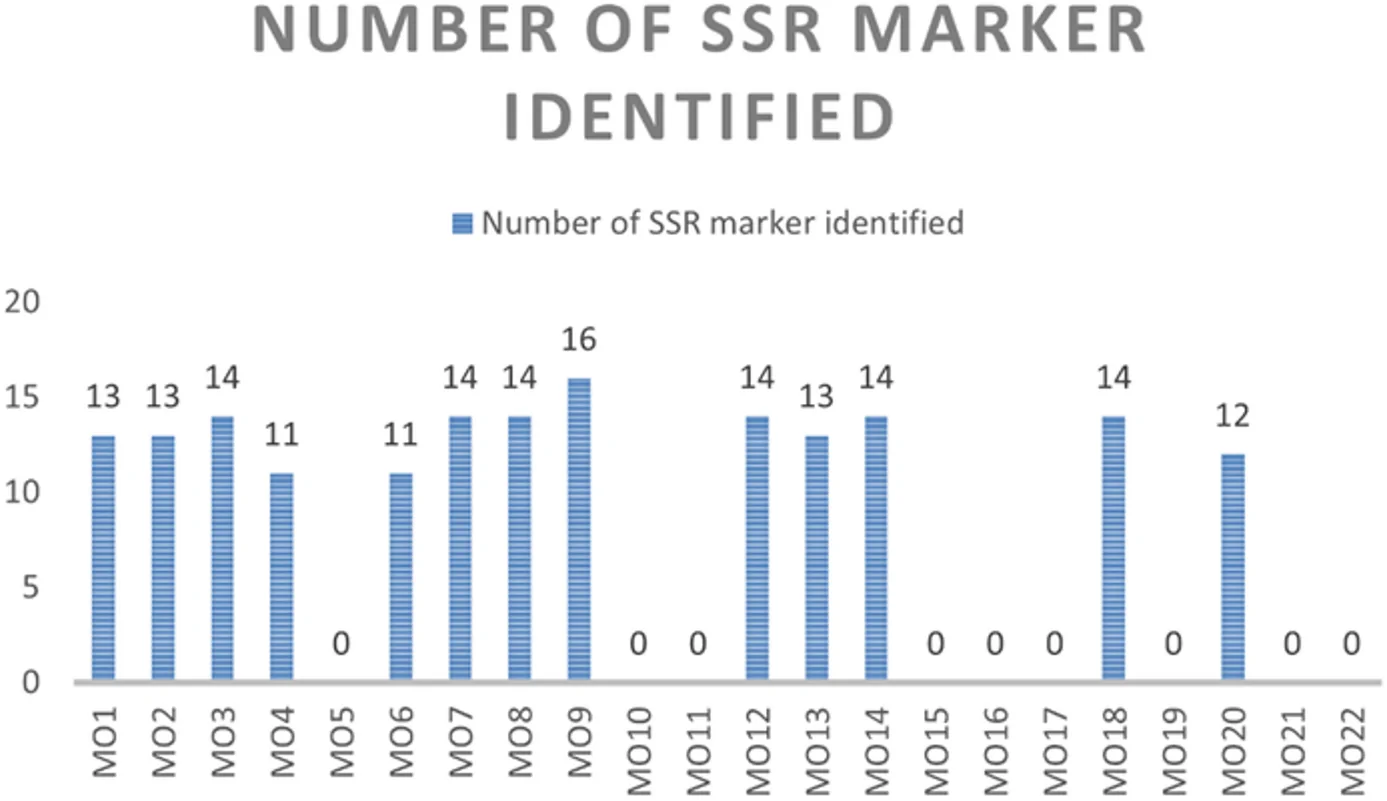
Distribution of SSR loci detected across the 22 *Magnaporthe oryzae* isolates, summarizing the number of markers identified per isolate using established microsatellite approaches.

The analysis of 22 *M. oryzae* isolates using 30 SSR markers revealed substantial genetic variability among the isolates. A total of 1–7 alleles were detected per marker, with several markers such as MGM12, MGM308, MGM401, MGM437, and MGM51 showing the highest allele numbers (5–7), indicating their high discriminatory power. The total number of loci amplified per marker varied widely from 1 to 16, reflecting differences in marker informativeness and genome coverage (Fig. 4).

Polymorphism Information Content (PIC) values ranged from 0 (monomorphic markers such as MGM266, Pyrms37_38, Pyrms43_44) to as high as 0.81 (MGM308 and MGM51), with most markers exhibiting moderate to high PIC (> 0.5), signifying good polymorphic potential for genetic diversity studies (Table 3).

**Table 3:** Genetic Diversity analysis showing Number of Alleles, Total number of loci and PIC (Polymorphism information content).

Sl. No.	SSR marker	Number of Alleles	Total number of loci	PIC (Polymorphism information content)
1.	MGM12	7	10	0.60
2.	MGM266	1	1	0
3.	MGM288	1	2	0
4.	MGM308	7	12	0.81
5.	MGM401	7	12	0.66
6.	MGM433	3	8	0.43
7.	MGM437	5	12	0.74
8.	MGM51	7	12	0.81
9.	Pyrms319_320	3	14	0.57
10.	Pyrms37_38	1	1	0
11.	Pyrms409_410	3	3	0
12.	Pyrms427_428	4	14	0.66
13.	Pyrms43_44	1	1	0
14.	Pyrms63_64	4	13	0.70
15.	Pyrms657_658	4	14	0.66
16.	Pyrms67_68	3	9	0.49
17.	Pyrms7_8	3	13	0.46
18.	Pyrms77_78	4	7	0.69
19.	Pyrms83_84	3	12	0.57
20.	Pyrms93_94	3	14	0.60

### Genetic relationships and clustering

#### Heatmap clustering

**Fig. 5:**
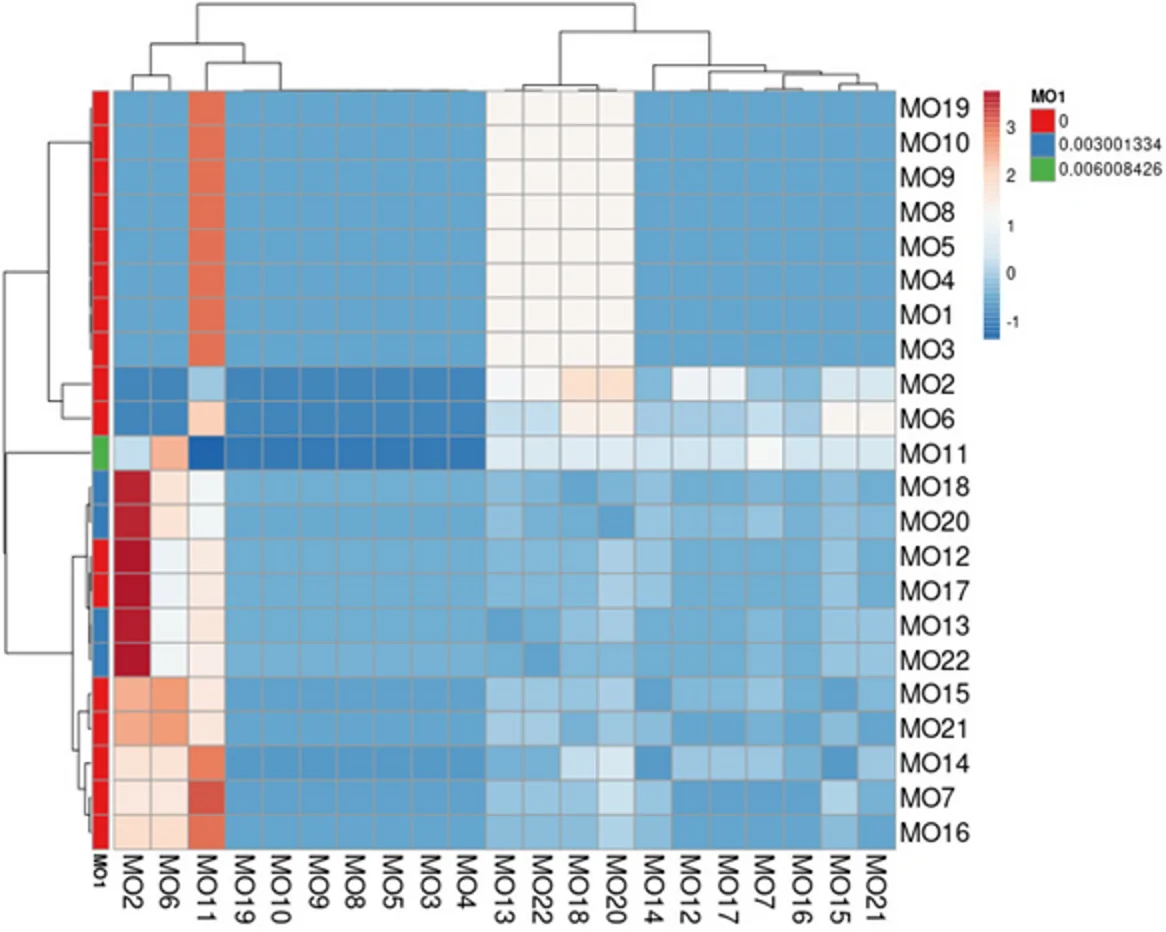
Heatmap of pairwise genetic distances among isolates. Blue shades indicate lower distances (greater genetic similarity), whereas red shades represent higher distances (greater divergence), following the standard interpretation of distance matrices.

The Genetic Distance Matrix Heatmap for 22 *M. oryzae* isolates (labelled MO1 to MO22), generated using 30 SSR (Simple Sequence Repeat) markers showed the colour scale on the heatmap representing genetic distance values, with blue colour tones indicating lower distances (higher similarity), and red colour tones indicating greater distances (lower similarity) (Fig. 5).

Most of the values appeared to cluster around very low distances (~ 0.00–0.04). Isolates MO2, MO6, MO11, MO13, MO18, MO20 and MO22 exhibited relatively higher genetic distances than several other isolates. MO3, MO4, MO5, MO8, MO9, MO10 and MO19 formed a cluster with almost no distance between them. MO13, MO18, MO20 and MO22 showed moderate diversity, but still tended to group in sub clusters with some isolates (Fig. 5). The cluster analysis identified two major genetic groups among the 22 *M. oryzae* isolates collected from Northeast India. Cluster I comprised 10 isolates‒MO19, MO10, MO9, MO8, MO5, MO4, MO1, MO3, MO2, and MO6‒and was further divided into two subclusters. Sub‒cluster I included eight isolates (MO19, MO10, MO9, MO8, MO5, MO4, MO1, and MO3), while Subcluster II contained two isolates (MO2 and MO6). Cluster II consisted of 12 isolates‒MO11, MO18, MO20, MO12, MO17, MO13, MO22, MO15, MO21, MO14, MO7, and MO16‒and was subdivided into three subclusters. Sub‒cluster I included only isolate MO11; Subcluster II comprised six isolates (MO18, MO20, MO12, MO17, MO13, and MO22); and Subcluster III consisted of five isolates (MO15, MO21, MO14, MO7, and MO16). A minimum distance of 0.0 indicated the presence of genetically identical samples, while the maximum distance of 0.1033 pointed to a few highly distinct pairs. The average pairwise distance (MPD) among isolates was 0.0087, showing a high degree of genetic similarity; the standard deviation of 0.0181 indicates some variability, with a few sample pairs showing greater divergence; and the Mean Nearest Taxon Distance (MNTD) of 0.0049 supported the observation that most isolates were closely related, with only a few outliers supporting to the overall diversity (Table 4).

**Table 4:** Genetic diversity statistics of 22 *Magnaporthe oryzae* isolates.

Metric	Value
Mean Pairwise Distance (MPD)	0.008729220014718616
Std Pairwise Distance	0.01813985938002251
Min Pairwise Distance	00
Max Pairwise Distance	0.1032990672
Mean Nearest Taxon Distance (MNTD)	0.004855420131818181

### Boxplot of pairwise genetic distances

**Fig. 6:**
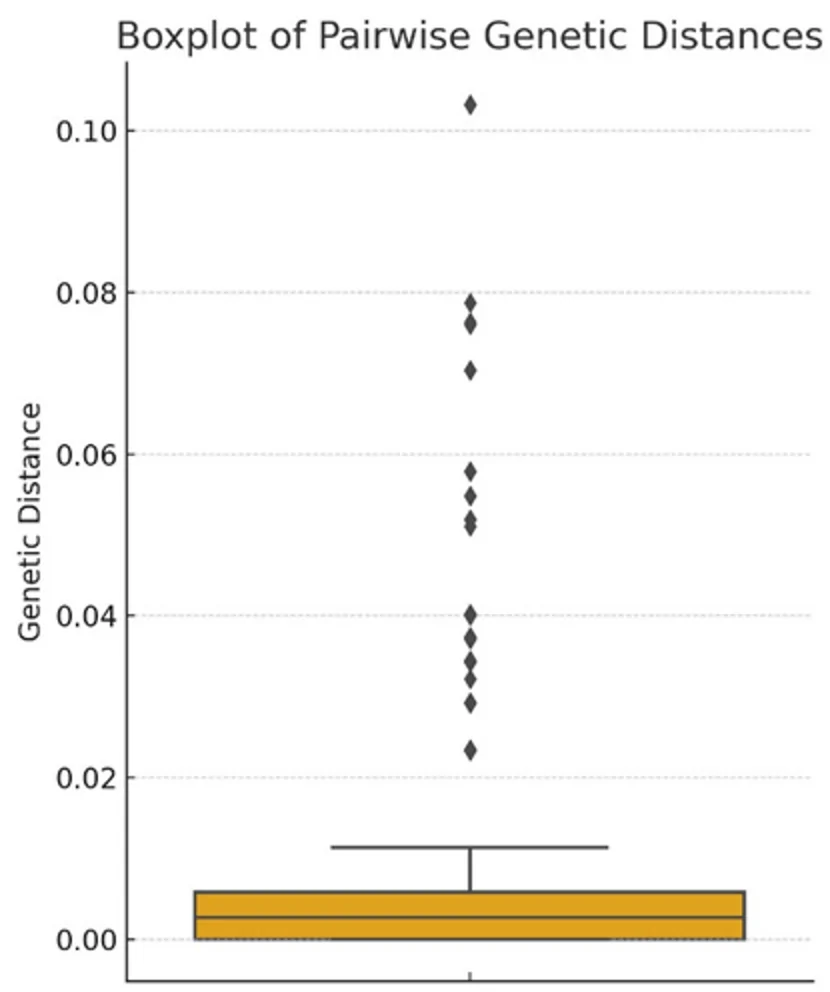
Boxplot displaying the distribution of pairwise genetic distances among the 22 *Magnaporthe oryzae* isolates from Northeast India, summarizing overall genetic relatedness.

The boxplot of pairwise genetic distances among 22 *M. oryzae* isolates showed that most genetic distances were clustered near zero. There were a relatively narrow interquartile range (IQR) and a low median value among these isolates. However, there was a presence of multiple outliers with higher genetic distances (ranging up to about 0.10) among the population (Fig. 6).

### Histogram of genetic distance frequencies

**Fig. 7:**
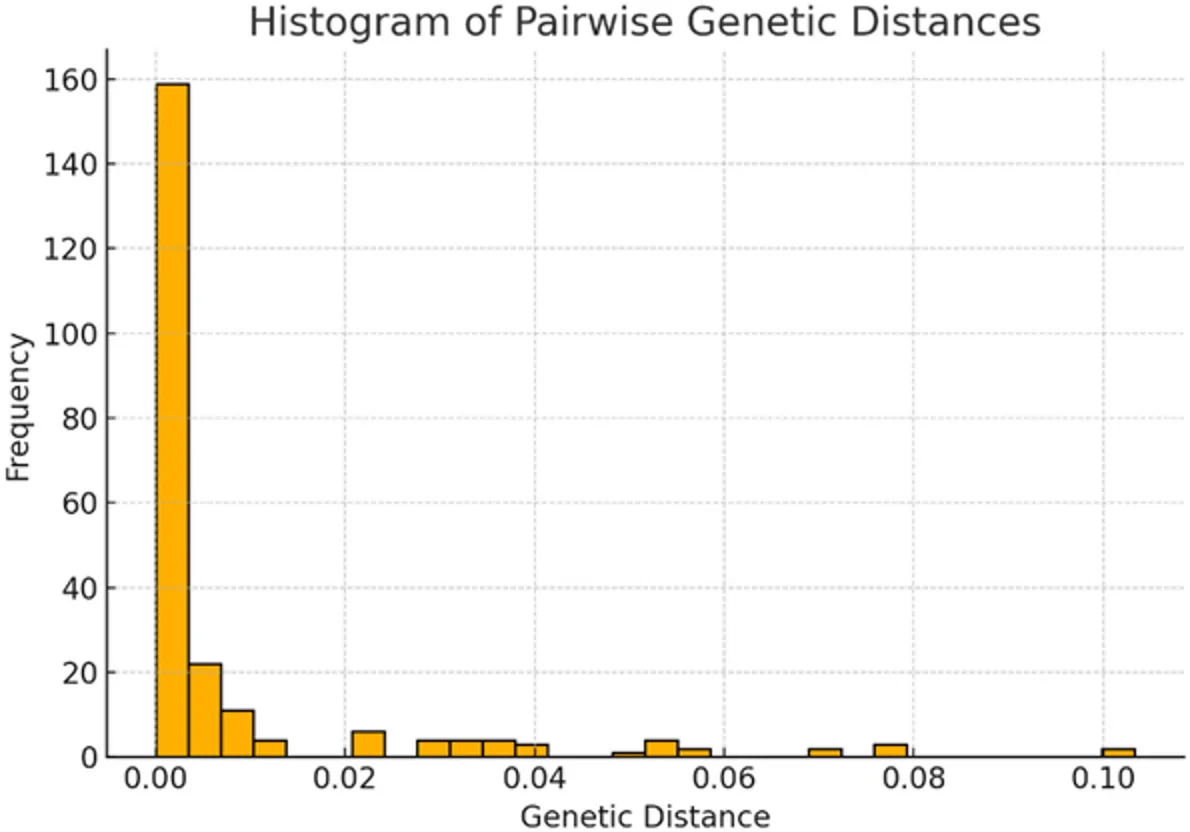
Histogram of pairwise genetic distances highlighting a strong skew toward very low values, confirming high genetic similarity among the isolates.

The histogram illustrated the distribution of pairwise genetic distances among 22 *M. oryzae* isolates. The majority of pairwise comparisons showed very low genetic distances (close to 0.00), while a smaller number of comparisons exhibited moderate to higher genetic distances (Fig. 7).

### Multivariate analysis (PCA and PCoA)

**Fig. 8:**
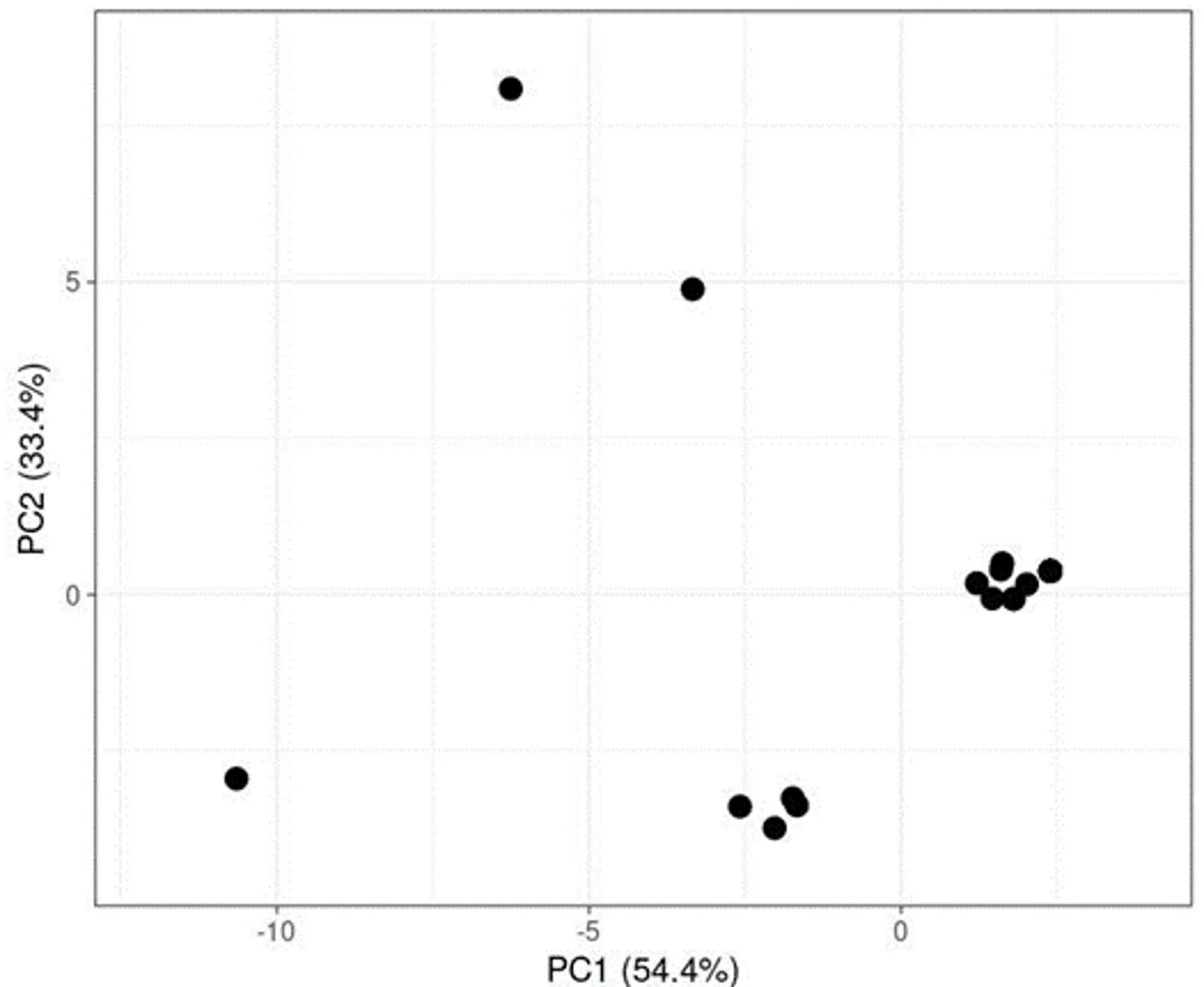
Principal Components Analysis (PCA) of the 22 *Magnaporthe oryzae* isolates, with axes representing variance explained by PC1 and PC2, illustrating clustering patterns typical of multivariate genetic analyses.

The 22 *M. oryzae* isolates showed significant genetic or phenotypic variation, as evidenced by their wide distribution across the plot. The first principal component (PC1) explains 54.4% of the total variance, and the second (PC2) explains 33.4%. Together, these two components accounted for 87.8% of the variation in the dataset (Fig. 8).

**Fig. 9:**
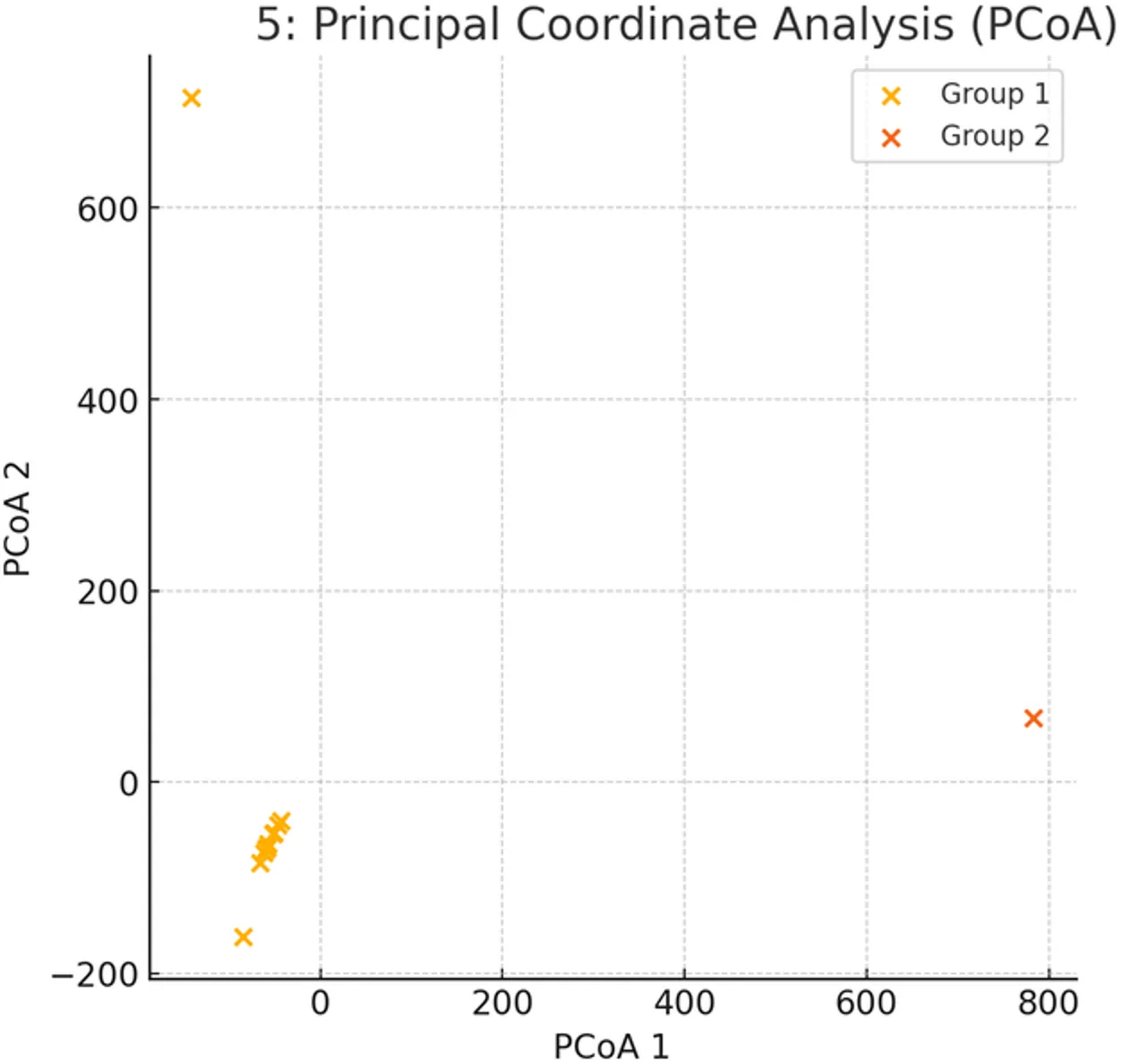
Principal Coordinate Analysis (PCoA) of 22 *Magnaporthe oryzae* isolates, showing how PCoA1 and PCoA2 capture the greatest proportion of variation within the dataset.

The PCoA 1 (X‒axis) and PCoA 2 (Y‒axis) represented the first two principal coordinates that explained the greatest amount of variation in the dataset. The magnitude of values along these axes reflected the genetic distances between isolates. Group 1 (yellow‒orange “x” markers): tightly clustered near the origin with small variations on both PCoA 1 and PCoA 2 axes. Group 2 (darker orange “x” markers): showed two outliers located far from the origin, especially on the PCoA 1 axis (~ 800) and PCoA 2 axis (~ 700) (Fig. 9).

### Population structure analysis by AMOVA

**Fig. 10:**
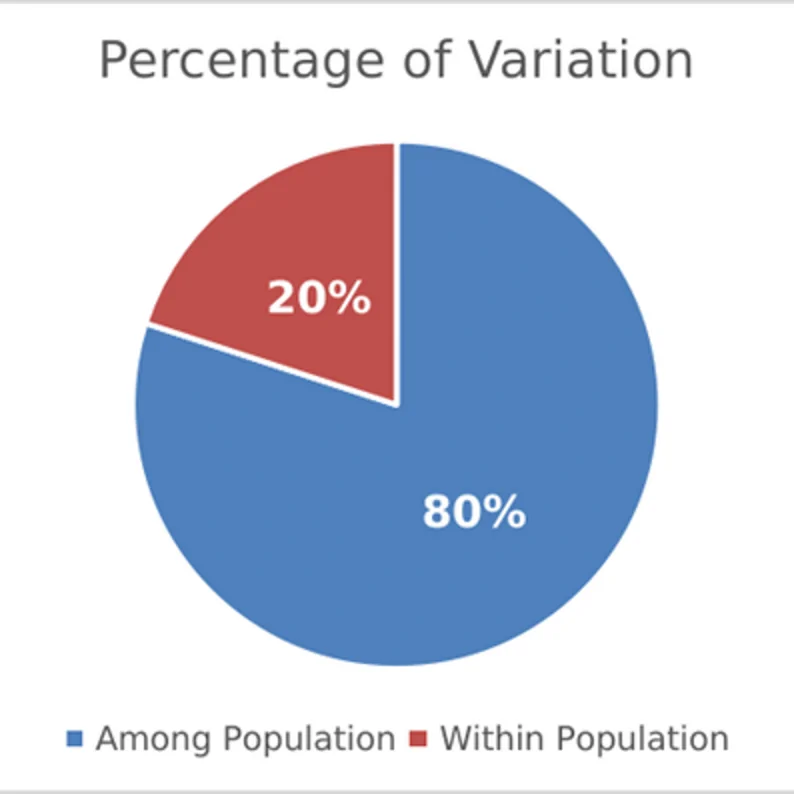
AMOVA partitioning of total genetic variation among the 22 *Magnaporthe oryzae* isolates into components representing variation among populations (states) and within populations.

The Analysis of Molecular Variance (AMOVA) of the 22 *M. oryzae* isolates collected from eight states in Northeast India partitioned the total genetic variation into two components: variation among populations (states) and variation within populations. States were treated as populations for AMOVA; however, uneven sampling may influence variance estimates. The result indicated that 80% of the total genetic variation was attributed to differences among populations, while only 20% occurred within populations (Fig. 10).

## Discussion

The 22 *M. oryzae* isolates were identified and characterised morphologically based on appearance on culture plates, colour of mycelium and growth rate. Our observed colony diameters of 36 mm to 90 mm, with a mean of 75.27 mm among the 22 isolates were partially matching with report of Jagadeesh and Devaki 2020^7^, in Karnataka, India, where the colony diameter range on PDA was reported as 80–90 mm for 15 isolates but the wider variations in the lower end (36 mm) is considerably lower. The study by Nazifa et al. 2021^8^ from Bangladesh on *M. oryzae* isolates, reported an average colony diameter of 50 mm on PDA for 28 isolates which is lower than our observed data. The limited sporulation and morphological uniformity observed among the 22 *M. oryzae* isolates (greyish white to whitish colonies, rapid growth, sparse sporulation) strongly suggest a genetically homogeneous population, likely arising from predominant clonal or asexual reproduction. Such homogeneity is commonly reported in field populations of *M. oryzae*, where genotyping and population structure studies have shown low genetic diversity and limited recombination, with most populations comprised of a few dominant lineages maintained largely through asexual reproduction.

The ITS‒based dendrogram of 22 *M. oryzae* isolates revealed considerable genetic relationship, with the isolates clustering into distinct groups. Sequencing of the ITS region of rDNA confirmed that all isolates belonged to *M. oryzae*, with most showing very high similarity (close to 100%) to reference sequences. A few isolates exhibited slightly lower intraspecific variation (95.5%–99.8% identity), suggesting limited genetic divergence within the studied population. Phylogenetic reconstruction using ITS sequences divided the isolates into two major clusters, reflecting the presence of genetically distinct subpopulations that possibly share common ancestry. The finding underscored the reliability of ITS sequencing for preliminary assessments of genetic relationship and for fungal species‒level identification. Results from this study are in agreement with regional analyses reported by Umakanth et al. 2017^9^ and Emani et al. 2024^10^, and align well with the phylogenetic observations of Palanna et al. 2023^11^. Branch lengths in the tree indicate varying degrees of genetic divergence. Some isolates (e.g., MO6–MO2 and MO13–MO14) showed very close similarity, while others, such as MO5 and MO18, represented more distant ancestries. Overall, the short branch lengths across the tree indicated that all isolates were genetically similar, consistent with variation expected within a single species.

Although the number of isolates per state was limited, the observed genetic structuring provides a preliminary understanding of population differentiation in the region. The existence of two major clusters emphasizes the internal diversity of the population, which may reflect virulence differences, potential gene flow, and underlying evolutionary processes. These insights are important for developing targeted disease management strategies and for guiding the selection of resistant rice genotypes in breeding programs. Overall, the topology of the phylogenetic tree suggests moderate genetic structuring, potentially shaped by factors such as geographic origin, host specialization, or restricted gene flow. Such phylogenetic analyses provided critical knowledge of *M. oryzae* evolution and supported informed approaches to disease management^12^.

Simple Sequence Repeat (SSR) markers are highly polymorphic and codominant, making them particularly effective for detecting fine‒scale genetic variation. Their resolution allows the identification of both closely related and genetically distinct isolates, confirming their utility in diversity and population studies^13^. In this study, 30 SSR markers were employed to genotype 22 *M. oryzae* isolates collected from Northeast India. A total of 74 alleles were identified, with allele numbers per locus ranging from 1 to 7. Markers such as MGM266, Pyrms37_38, and Pyrms43_44 were monomorphic, while MGM12, MGM308, MGM401, and MGM51 exhibited up to seven alleles, demonstrating substantial allelic richness. A value of zero SSR loci in isolates like MO5, MO10, MO11, MO15, and MO16 most likely reflect amplification failure probably due to one or multiple reasons such as presence of null alleles, caused by mutations in primer‒binding regions that prevent primer annealing, technical PCR issues‒poor DNA quality, inhibitors, or suboptimal PCR conditions that can lead to no detectable bands, rather than true absence of SSRs, which are generally abundant in genomes.

Polymorphism Information Content (PIC) values further reflected the informativeness of the markers, ranging from 0.0 at monomorphic loci to 0.81 for highly polymorphic ones (e.g., MGM308 and MGM51). The average PIC value across all loci was 0.55, suggesting a moderate to high level of polymorphism. Markers with PIC values above 0.7 (e.g., MGM308, MGM51, MGM437, and Pyrms63_64) were especially informative for population genetics and diversity assessments, whereas monomorphic markers offered little resolution. High PIC markers are therefore particularly useful for analyzing population structure, monitoring virulence, and guiding resistance breeding programs. These findings are consistent with earlier studies of *M. oryzae* populations in Karnataka^7^ and other parts of India^14^.

The heatmap displayed the genetic distance matrix among 22 *M. oryzae* isolates, labeled MO1‒MO22. The colour gradient represented genetic similarity, with blue tones indicating lower genetic distances (higher similarity) and red tones indicating higher genetic distances (greater divergence). Hierarchical clustering along both axes grouped the isolates with similar genetic profiles, revealing the presence of both high genetic similarity and some divergent genotypes. Most isolates clustered closely, forming predominantly blue‒shaded matrices that reflected genetic homogeneity. However, isolates such as MO1, MO2, M03, MO6 and MO11 displayed shades of red (values > 0.10), indicating greater divergence when compared to several isolates like MO3, MO4, and MO5. The cluster analysis in this study revealed two major genetic groups among the Northeast Indian *M. oryzae* isolates, indicating a moderate but significant population subdivision. However, the presence of divergent outliers (e.g., MO2 and MO11) indicated occasional introductions of novel genotypes through mutation, recombination, or geographical migration also reported by Latorre et al. 2023^15^. This combination of overall genetic uniformity with pockets of divergence has important implications: high diversity markers such as MGM308 and MGM51 can be leveraged for population monitoring, while recognition of genetically distinct isolates is essential for anticipating pathogen evolution, maintaining durable resistance, and guiding rice blast management strategies. Such outliers represent a direct threat to durable resistance, as they may carry virulence genes that can overcome commonly deployed resistance (R) genes^16^.

Overall, the heatmap demonstrated substantial genetic diversity within the *M. oryzae* population. This finding aligns with the high genetic diversity reported in the putative center of origin in Southeast Asia^17^, yet reflects a more structured population than might be expected from a purely clonal pathogen. The prevalence of a genetically homogeneous majority, interspersed with highly divergent isolates like MO2 and MO13, suggests a population influenced by both asexual propagation and occasional recombination or migration events. The observed genetic homogeneity is consistent with predominantly clonal reproduction, as reported in other *M. oryzae* populations. Similar trends‒large homogeneous clusters interspersed with rare, divergent genotypes have been reported in *M. oryzae* populations across India, Brazil, Colombia, Thailand, and elsewhere, shaped by factors such as historical introductions, spore dispersal, seed exchange, and local adaptation^7,10,18–21^. Metrics such as mean pairwise distance (MPD) and mean nearest taxon distance (MNTD) further supported the conclusion of a genetically narrow population, likely driven by the recurrent use of genetically uniform rice varieties. Nonetheless, outliers such as MO20 suggest possible introductions from external populations or the emergence of novel variants through mutation or adaptation.

The boxplot of pairwise genetic distances among 22 *M. oryzae* isolates shows that most genetic distances are clustered near zero, indicating a high level of genetic similarity within the population. The relatively narrow interquartile range (IQR) and low median value suggest limited overall genetic variation among these isolates. However, the presence of multiple outliers with higher genetic distances (ranging up to about 0.10) suggests that a few isolates exhibit greater genetic divergence, potentially representing distinct lineages or unique evolutionary events within the population. The histogram of genetic distances of 22 isolates confirmed this pattern, showing a strong skew toward low values and a prominent peak near zero, consistent with clonal reproduction and limited genetic exchange^22^. Occasional higher values underscored the presence of genetically distinct isolates that contribute to overall diversity and could drive future outbreaks.

Principal Component Analysis (PCA) for the 22 Northeast Indian *M. oryzae* populations revealed that PC1 and PC2 accounted for 54.4% and 33.4% of the total variance, respectively, together explaining 87.8% of the dataset’s variation. This high explanatory power indicates that the two‒dimensional projection effectively captured genetic differences among the fungal isolates. While some isolates formed tight clusters, others were spread along the PC1 axis, suggesting substantial diversity. The separation of isolates into distinct subgroups may be linked to geographic origin, host specificity, or virulence traits. Principal Coordinates Analysis (PCoA) supported these findings. Group 1 isolates, represented near the origin, formed a dense cluster indicative of high similarity and possible recent common ancestry. In contrast, Group 2 contained two highly divergent isolates, positioned far along both coordinate axes, reflecting substantial differentiation. This structuring suggests that Group 1 represents a clonal or recently diverged lineage, while Group 2 harbours novel or recombinant genotypes with potentially greater adaptability. Such divergence increases the evolutionary potential of the pathogen, posing significant challenges for resistance management. Overall, both PCA and PCoA demonstrate significant genetic diversity within *M. oryzae* populations from Northeast India. Similar genetic structuring, characterized by high intra‒group similarity and inter‒group divergence, has been documented in other regional studies^18,19^.

The Analysis of Molecular Variance (AMOVA) of the 22 *M. oryzae* isolates collected from eight states in Northeast India revealed a high proportion of among population variation suggesting significant genetic differentiation and limited gene flow among the fungal populations in the region, possibly due to geographical isolation, environmental variation, cultivation of diverse land races or localized adaptation. At the same time, the presence of genetically divergent isolates such as MO2 and MO11 highlights the risk of resistance breakdown in rice cultivars, as these variants may be better equipped to overcome host defenses. Similar patterns of population subdivision linked to geography and environment have been reported in other regional and international studies^7,19^. Consequently, *M. oryzae* populations in the Northeast appear to be highly structured geographically, reflecting distinct evolutionary or adaptive lineages across the states. The predominance of a clonal population structure with high genetic similarity among *M. oryzae* isolates in Northeast India indicates the widespread circulation of a few successful pathogen lineages, leading to predictable but potentially severe blast epidemics under favourable conditions. The presence of few genetically distinct outliers suggests a risk of emerging virulent strains that could overcome existing host resistance. Epidemiologically, this highlights the importance of continuous surveillance, while for resistance breeding it supports targeting dominant pathogen clones through effective resistance genes combined with gene pyramiding to ensure durable blast resistance in Northeast Indian rice varieties.

## Conclusion

The isolates of the rice blast pathogen in Northeast India exhibits pronounced morphological variation and a markedly structured, predominantly clonal population. Substantial genetic diversity reflected in multiple ITS sequence types, high SSR polymorphism, and strong differentiation among populations‒highlights the complexity of pathogen dynamics across the region. These findings highlight considerable genetic diversity among *M. oryzae* isolates from Northeast India, underscoring the need for continuous monitoring and adopting integrated management practices. Incorporating a wide spectrum of resistance genes in breeding programs and diversifying cultivation strategies will help mitigate selection pressure and delay resistance breakdown.

## Electronic Supplementary Material

Below is the link to the electronic supplementary material.

## Data Availability

The datasets (22 paired FASTQ files) generated and analysed in this study are available in the NCBI Sequence Read Archive (SRA) under accession number PRJNA1432504 (https://www.ncbi.nlm.nih.gov/sra/PRJNA1432504).
