# Supplementary material for: Morphological characterization, genetic diversity and population structure of the rice blast pathogen Magnaporthe oryzae in Northeast India
Source: Sci Rep. 2026 Jun 30;16:27539. doi: 10.1038/s41598-026-51508-9 (PMC13538464; doi:10.1038/s41598-026-51508-9)
Supplement: Supplementary file 1 [file 41598_2026_51508_MOESM1_ESM.docx]

| **Supplementary Table 1. List of SSR markers used in the study.** | | | | |
| --- | --- | --- | --- | --- |
| **Sl. No.** | **Marker** | **Primer sequences**  Forward | **Primer sequences**  Reverse | **T(°C)** |
| 1. | MGM12 | TGATTGCGTGATCCT TTCAG | CTCTTTTCGCCAAGA | 58 |
| 2. | MGM51 | GTAACCAGGCCGTTT CAAGA | GGAGGTTGCAGA | 60 |
| 3. | MGM266 | TGTGGTGGGTGATCT TGTTG | ATTCCCGGCGAG | 62 |
| 4. | MGM286 | CGGCTGTGGTTTAAC GATTT | CCATCAGGATCCATG | 58 |
| 5. | MGM288 | TTGTCGACGAGTGTC CAAAG | CAGTTACCCCGTCGG | 60 |
| 6. | MGM308 | TTGTCAGGCAGGACC ATGTA | TTGTGTCTGCCTTGC | 58 |
| 7. | MGM401 | TCCATGAGAGACGGT TTCAA | GTGGTCCCCGTTGTG | 58 |
| 8. | MGM433 | CCTGGCCCAAGCATT ATCTA | ACTTGAAGGGCC | 58 |
| 9. | MGM437 | GCCCCTCAATAGATC GTCAA | ACTGCGGCATTTTAA | 60 |
| 10. | Pyrms 07-08 | GCAAATAACAGGAAAACG | AGAAAGAGACAAAACACTGG | 53 |
| 11. | Pyrms 37-38 | ACCCTACCCCACTCATTTC | AAGGATCAGCCAATGCCAAGT | 55 |
| 12. | Pyrms 39-40 | AGGATCAGCCAATGCCAAGT | CGCATACAGGAAAGCCAAGA | 53 |
| 13. | Pyrms 43-44 | TCAGTAGGCTTGGAATTGAAAAA | CTTGATTGGTGGTGGTGTTG | 55 |
| 14. | Pyrms 47-48 | TCACATTTGCTTGCTGGAGT | AGACAGGGTTGACGGCTAAA | 55 |
| 15. | Pyrms 63-64 | TTGGGATCTTCGGTAAGACG | GCCGACAAGACACTGAATGA | 55 |
| 16. | Pyrms 67-68 | AGCAAGCAGGAGATGCAGAC | GTTTGGCTGGCAAGACAGTT | 55 |
| 17. | Pyrms 77-78 | GAAGTATTGCACACAAACAC | GCTTTCGGCAAGCCTAATC | 55 |
| 18. | Pyrms 83-84 | GTCTGCCTCGACTCCTTCAC | AGCCCAAAAACAGAAAGCAA | 55 |
| 19. | Pyrms 87-88 | AGACTTGTTACTCGGGTCTTGA | CCAGATGTCACTCCCCTGTA | 53 |
| 20. | Pyrms 93-94 | CCTCGACTCCTTCACCAAAA | CGGAGAGCTCAGGAAGAGG | 55 |
| 21. | Pyrms 99-100 | CACCACTTTATGGCGCAGT | ACCTAGGTAGGTATACATGTTGTT | 55 |
| 22. | Pyrms 101-102 | CTGCGTTCAACATGCCTCTA | CTTAGATCTGCGGTATGAGCA | 55 |
| 23. | Pyrms 233-234 | TGAGATGGACCGCATGATTA | TTGATGGCAGAGACATGTAGC | 55 |
| 24. | Pyrms 319-320 | TAAGACCACTGGCGGAATCT | GGCTTTGTCTGGTTGTACGG | 55 |
| 25. | Pyrms 409-410 | TCCCAGTACTTGCCCATCTC | ATCTCATATCCGTCGGTCGT | 55 |
| 26. | Pyrms 427-428 | CTGTCACCACAACCAAGACG | TTGCCCTGATTTGTCAGTCA | 55 |
| 27. | Pyrms 505-506 | CCTGTCGTAGCACCTTTGGT | TTGTTCCTCGTCCCATGTTT | 55 |
| 28. | Pyrms 533-534 | CTTATCGGGAGGTGCAGAAG | GACGGCATACTGCATACGTG | 55 |
| 29. | Pyrms 607-608 | CCCAAGCTCCATAATAGGCTAC | TCCGAGACTCTTTGGATAGCAC | 55 |
| 30. | Pyrms 657-658 | ATCAGTCGAACCCACAAAGC | ATGTGTGGACGAACCAGTCC | 55 |
